# Design and test of a rigid endomicroscopic system for multimodal imaging and femtosecond laser ablation

**DOI:** 10.1117/1.JBO.28.6.066004

**Published:** 2023-06-28

**Authors:** Chenting Lai, Matteo Calvarese, Karl Reichwald, Hyeonsoo Bae, Mohammadsadegh Vafaeinezhad, Tobias Meyer-Zedler, Franziska Hoffmann, Anna Mühlig, Tino Eidam, Fabian Stutzki, Bernhard Messerschmidt, Herbert Gross, Michael Schmitt, Orlando Guntinas-Lichius, Jürgen Popp

**Affiliations:** a GRINTECH GmbH, Jena, Germany; b Leibniz Institute of Photonic Technology, Member of Leibniz Health Technologies, Member of the Leibniz Centre for Photonics in Infection Research, Jena, Germany; c Friedrich Schiller University Jena, Institute of Physical Chemistry and Abbe Center of Photonics, Member of the Leibniz Centre for Photonics in Infection Research, Jena, Germany; d Jena University Hospital, Department of Otorhinolaryngology, Jena, Germany; e Active Fiber Systems GmbH, Jena, Germany; f Fraunhofer Institute for Applied Optics and Precision Engineering, Jena, Germany

**Keywords:** nonlinear imaging, optical biopsy, endomicroscope, indocyanine green imaging, microsurgery probe, tissue ablation

## Abstract

**Significance:**

Conventional diagnosis of laryngeal cancer is normally made by a combination of endoscopic examination, a subsequent biopsy, and histopathology, but this requires several days and unnecessary biopsies can increase pathologist workload. Nonlinear imaging implemented through endoscopy can shorten this diagnosis time, and localize the margin of the cancerous area with high resolution.

**Aim:**

Develop a rigid endomicroscope for the head and neck region, aiming for *in-vivo* multimodal imaging with a large field of view (FOV) and tissue ablation.

**Approach:**

Three nonlinear imaging modalities, which are coherent anti-Stokes Raman scattering, two-photon excitation fluorescence, and second harmonic generation, as well as the single photon fluorescence of indocyanine green, are applied for multimodal endomicroscopic imaging. High-energy femtosecond laser pulses are transmitted for tissue ablation.

**Results:**

This endomicroscopic system consists of two major parts, one is the rigid endomicroscopic tube 250 mm in length and 6 mm in diameter, and the other is the scan-head ($10\times 12\times 6\;{\mathrm{cm}}^{3}$ in size) for quasi-static scanning imaging. The final multimodal image accomplishes a maximum FOV up to 650  μm, and a resolution of 1  μm is achieved over 560  μm FOV. The optics can easily guide sub-picosecond pulses for ablation.

**Conclusions:**

The system exhibits large potential for helping real-time tissue diagnosis in surgery, by providing histological tissue information with a large FOV and high resolution, label-free. By guiding high-energy fs laser pulses, the system is even able to remove suspicious tissue areas, as has been shown for thin tissue sections in this study.

## 1 Introduction

As the vital organ for breathing, speaking, and swallowing, the healthy condition of the larynx can influence our quality of life significantly. Due to high consumption of tobacco, laryngeal cancer is a common tumor of the respiratory tract.^1^ A rapid intraoperative diagnosis of it can be established by a frozen section procedure on the suspicious mass, but its results can be inaccurate. Accurate diagnosis relies on histopathological analysis of the formalin-fixed paraffin-embedded process biopsy, which requires several days. “Optical biopsy” as an emerging technique, aiming to provide diagnostic signatures *in-situ*, noninvasively and in real-time, can assist surgeons in decision-making and reduce pathologist workload.^2^ For example, multimodal nonlinear imaging, including coherent anti-Stokes Raman scattering (CARS), two-photon excitation fluorescence (TPEF), and second harmonic generation (SHG), is capable of providing morphochemical contrast, without the need of exogenous markers.^3^^,^^4^ A prediction accuracy of 90% can be achieved for diagnosing imaged tissue sections.^5^ Apart from that, indocyanine green (ICG) is one of two U.S. Food and Drug Administration approved exogenous contrast agents for clinical use. It has peak absorption and emission wavelengths in the near-infrared spectrum and offers contrast for vascular structures. Moreover, combining the optical biopsy with fs laser ablation can be potentially useful for immediate and high precision ablation of cancerous tissue.^6^

Over the past decade, substantial progress has been made in the development of flexible or rigid endomicroscopes for *in-vivo* nonlinear imaging. By employing a fiber-based ultrafast laser source, the entire device can be moderated to a compact size convenient for transportation. Hollow core fiber and double core fiber are at present two solutions for laser delivery,^7^^,^^8^ because both of them suppress the four wave mixing background generated by two excitation beams of CARS in a glass fiber core.^9^ For the scanning mechanism, piezoelectric actuator or scanning mirrors based on micro-electro-mechanical systems (MEMS) are mostly implemented for a miniaturized endomicroscope,^8^^,^^10^^–^^14^ as both of them exhibit great flexibility in a limited assembly space. However, such micro-scanners have are limited by the scanning angle^15^ and increase the difficulty in assembly and sterilization. For imaging certain organs, e.g., in the head and neck region, where miniaturized distal tip is not necessary, proximal scanning by a galvanometer (Galvo) scanner is more advantageous.^16^ This scanner is also much easier to be mounted than micro-scanners due to its bulky size, and offers uniform scanning pattern in the quasi-static mode. Besides, by comparison with the MEMS mirror, Galvo scanner achieves a large tilt angle while still employing a large size scanning mirror, both of which are beneficial for increasing the field of view (FOV) and numerical aperture (NA) on the image plane. Most of the published endomicroscopes using nonlinear imaging techniques achieved ∼1  μm lateral resolution owing to high NA light focused by the objective lens. This also generates a short depth of focus, which however can lead to field curvature effects dominant at the field edge. Although a slightly curved field plane can be tolerated when imaging in-vivo tissues, it becomes problematic for measuring thin tissue sections in *ex-vivo*. Regarding femtosecond (fs) laser surgery, so far the main field in which this technique has been successfully introduced into the clinical setting is ophthalmology, with fs-LASIK^17^ being the most famous case. However, in recent years, many researchers have shown the potential of the technique for different clinical applications.^6^^,^^18^ To date, only very few endomicroscopic systems are designed for simultaneous nonlinear imaging and tissue ablation by fs laser, and the performance is still limited in terms of the FOV and physical dimension, for example.^19^ Therefore, an increase of FOV with a reduced probe size would be advantageous.

In this article, we demonstrate a new multimodal nonlinear endomicroscopic device for the upper aerodigestive tract. The device can be hand-held and comprises one rigid endomicroscopic tube (250 mm length, 6 mm outer diameter) and one scan-head ($10\times 12\times 6\;{\mathrm{cm}}^{3}$ in size). A 3 mm collimated beam is scanned by a two-axis Galvo scanner and focused with NA of 0.5 on the tissue side, achieving a maximum FOV of 650  μm. To our knowledge, this is the largest FOV with such high NA for endomicroscopic imaging systems.^20^^,^^21^ The endomicroscopic tube has corrected field curvature and color aberration from 800 to 1030 nm in the optical design. Two signal collection modes are designed in the scan-head. One is arranged in the non-descanned way for collecting nonlinear signals, i.e., CARS/TPEF/SHG, from the sample. The other is arranged for collecting single photon fluorescence of ICG in confocal detection. All intermediate foci are in air and all glass surfaces are illuminated by a laser beam larger than 1 mm in diameter. Therefore, high-energy fs pulse of up to μJ energy level can be guided without glass damage, and complete tissue ablation at the distal end with high precision.

## 2 Design Concept and Simulation

### 2.1 Scan-Head and the Endomicroscopic Tube

**Fig. 1:**
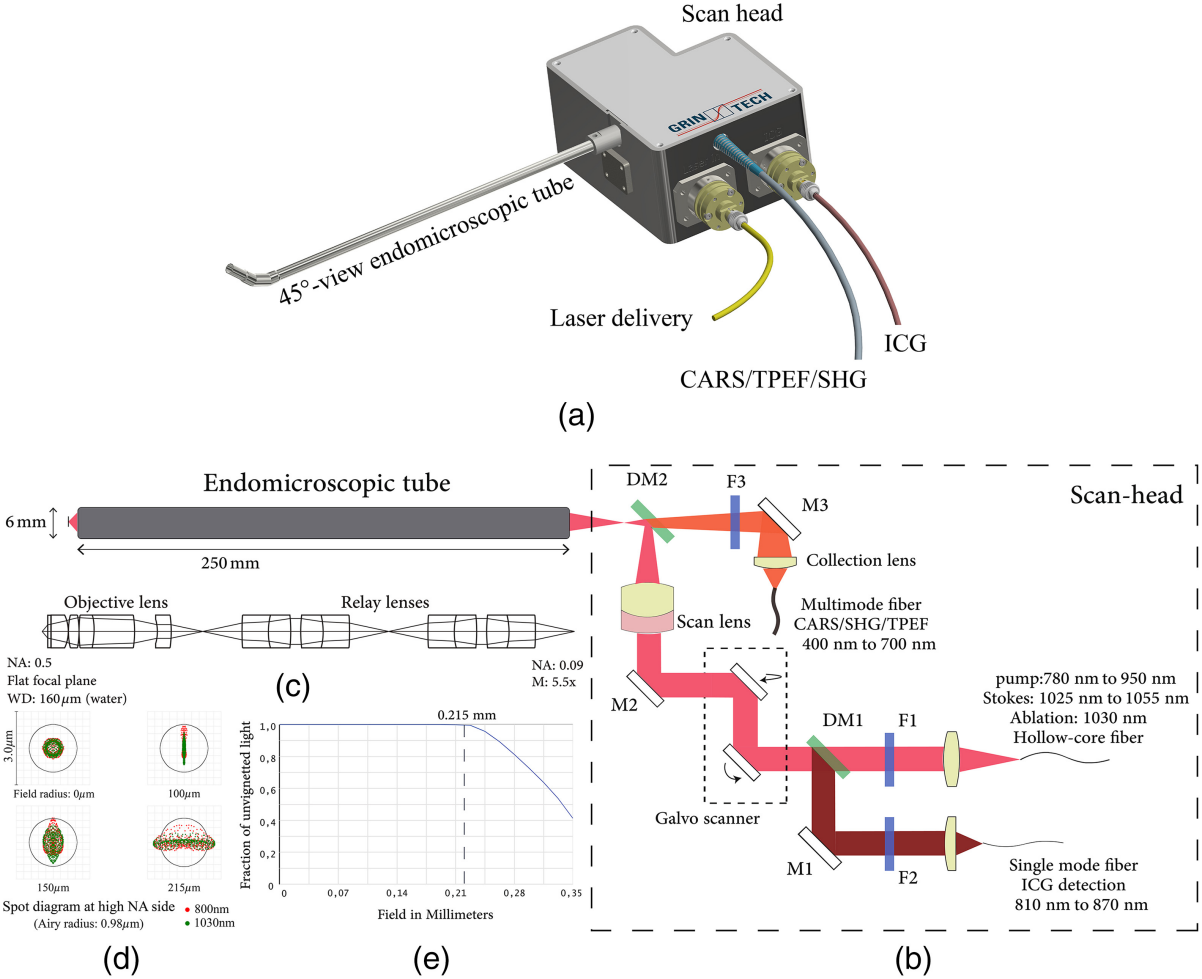
(a) Overview of the whole device (excitation laser, PMT detection unit, and scanner controller are not included). (b) Optical path of the system. F1: filter LP750, F2: BP842/56, F3: filter SP750, DM1: dichroic mirror ZT845/42, DM2: dichroic mirror SP749, M1: dielectric coating mirror, M2: silver coating mirror, and M3: silver coating mirror. (c) Optical layout of the endomicroscopic tube. (d) Spot diagram of the endomicroscopic tube at different fields of two wavelengths. Red and green represent 800 nm and 1030 nm. Airy radius is 0.98  μm for NA=0.5. (e) Vignetting diagram for field radius from 0  μm to 350  μm.

Figure 1(a) shows an overview of the endomicroscopic device.^22^ The scan-head and the endomicroscopic tube are connected by a robust flange, and the rigid endomicroscopic tube is developed in two variants: 0 deg-view and 45 deg-view. The 45 deg-view tube is shown in the picture. As illustrated by the optical path in Fig. 1(b),^23^ excitation beams are delivered together from a hollow-core fiber (PMC-C-R&D, GLOphotonics), and first collimated by a custom-made Galilean telescope (f=60  mm) then deflected by a Galvo scanner (Cambridge Technology, 6210H). A long-pass filter F1 (FELH0750, Thorlabs) removes four-wave mixing noise from the delivery fiber. A silver mirror M2 (112622, Layertec), a scan lens (f=16.67  mm), and a short-pass dichroic mirror DM2 (FF749, Semrock) reflect and focus the beam into the endomicroscopic tube. Since nonlinear signals are within the visible wavelength range, part of them propagate back and pass the dichroic mirror DM2. After a short-pass filter F3 (FESH0750, Thorlabs) for blocking the near-infrared light, a custom-made telescope lens (magnification: 3.75×) conjugates nonlinear signals to the multimode fiber surface (M107L02, Thorlabs). ICG signals ranging in 810 to 870 nm are transmitted back along the excitation optical path until being reflected by a notch-band dichroic mirror DM1 (ZT845/20, Chroma). They are finally coupled in a single mode (SM) fiber (P1-780A-FC-5, Thorlabs) by a doublet lens (60SMS-M12, Schäfter + Kirchhoff).

Both variants of the endomicroscopic tube use same lenses except an additional prism used in the 45 deg-view tube. The optical layout in Fig. 1(c) is the 0 deg-view tube. A custom-made objective lens and two units of the custom-made relay lenses (5 mm in diameter), as well as a steel housing (25 cm in length and 6 mm in diameter) constitute the long rigid tube. Inside the tube, all intermediate foci locate at least 5.5 mm in distance from the glass surfaces. To mimic the working medium in the tissue, the working space of tube’s distal end is designed in water, and its working distance is 160  μm. The NA of the endomicroscope is 0.09 on the proximal side close to the scan-head and 0.5 at the distal end. To generate such a high NA, the front component in the objective lens has an aplanatic lens surface, which helps to enlarge the NA without introducing spherical aberration.^24^ On the other hand, the field curvature becomes critical in large FOV, hence a meniscus lens is utilized to correct this optical aberration so that the focal plane is flat.^25^ Figure 1(d) shows the spot diagram at the high NA side of the endomicroscopic tube. Operation wavelengths 800 and 1030 nm are indicated in red and green. The black framed circle indicates the Airy disk for NA 0.5. From the field radius 0 to 215  μm, spot sizes are below the diffraction limit. Meanwhile, two wavelength patterns corresponding to the pump and Stokes beams for CARS excitation overlap each other both in the on-axis and off-axis fields, which mean the axial and lateral chromatic aberrations of the first order are corrected. Additionally, according to the energy throughput diagram in Fig. 1(e), the endomicroscopic tube starts to get vignetted from the field radius of 215  μm, and suffers from 60% energy loss as the field rises to 350  μm off-axis.

### 2.2 Nonlinear Signal Collection by the Multimode Fiber

**Fig. 2:**
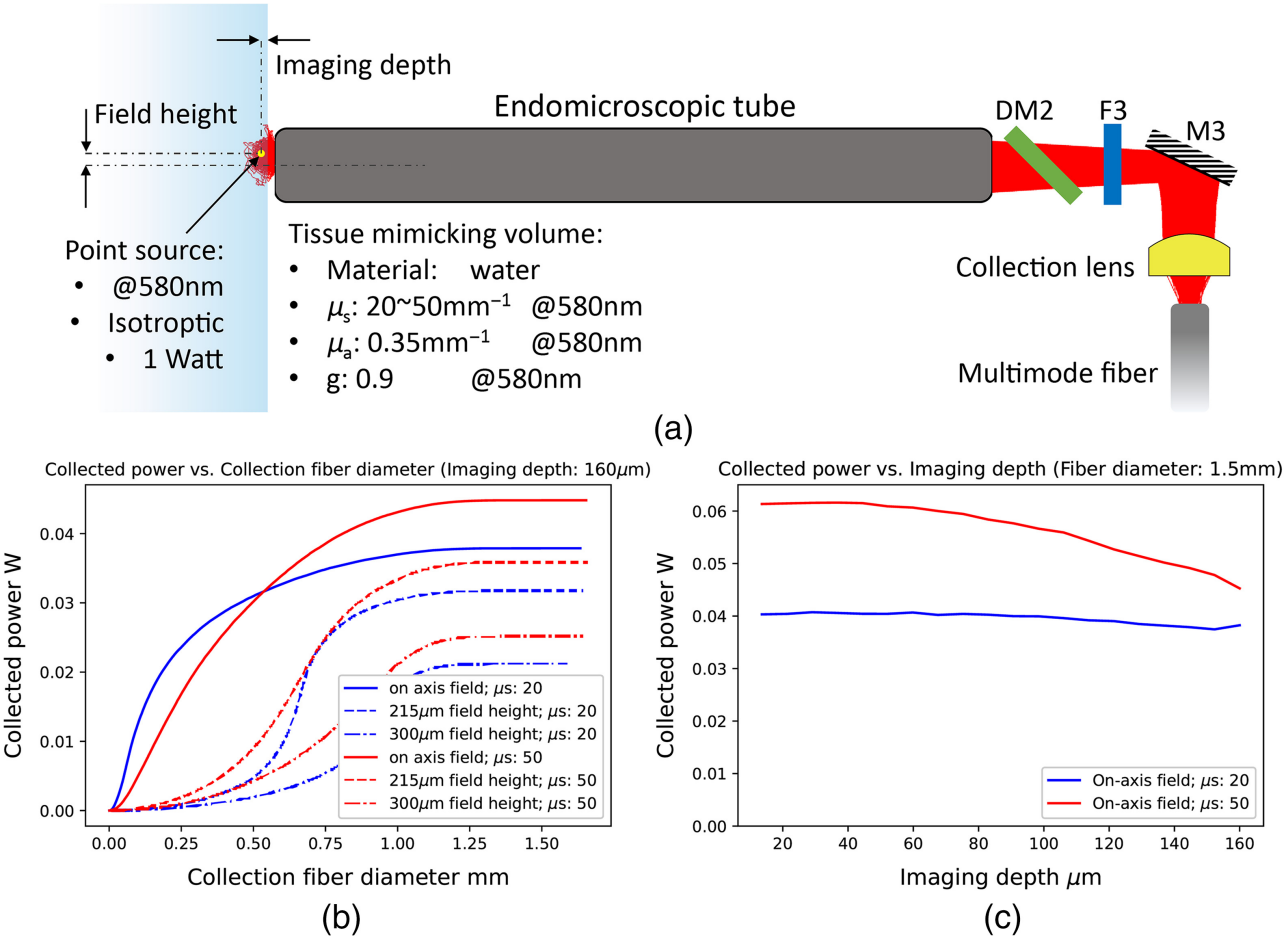
(a) Schematic diagram of the non-descanned collection channel. Light scattering in the tissue-mimicking volume is also drawn. The point source represents the emission point of the nonlinear signals. The field height is a distance of the source to the optical axis. The imaging depth relates to the distance from the point source to the volume surface. (b) The collected power for different sizes of the collection fiber. Imaging depth was set to 160  μm. Blue and red lines represent results when $\mu_{s}$ is $20\;{\mathrm{mm}}^{-1}$ and $50\;{\mathrm{mm}}^{-1}$. Solid, dash, and dot-dash lines represent results when field heights are on-axis, 215  μm and 300  μm. (c) The collected power in different imaging depths. The multimodal fiber core was set 1.5 mm in diameter. Blue and red indicates two cases of the scattering coefficients $20\;{\mathrm{mm}}^{-1}$ and $50\;{\mathrm{mm}}^{-1}$, respectively.

Unlike single photon excitation imaging, nonlinear signals only come from the excited focal volume.^26^ However, signals get strongly scattered in the tissue, which leads to a reduction of the final collection efficiency. Figure 2(a) illustrates the collection of the scattered signal by a multimode fiber through the non-descanned detection channel. To collect maximum signals from the excited area, an appropriate fiber core and acceptance NA need to be selected. Hence, a simulation was performed to investigate the collected power for different scattering properties, field heights, and imaging depths. The ray-tracing process was operated by the non-sequential mode in the optical design and simulation software OpticStudio. Light scattered in the volume originated from a point source (marked in yellow) localized at the position, where the excitation light was focused at a certain field height. The point source generated an isotropic radiation at 580 nm with 1 W in total power, and 1,000,000 rays were traced in OpticStudio. The tissue-mimicking volume filled in with water was modeled by three scattering factors: (1) the scattering coefficient ($\mu_{s}$), (2) the absorption coefficient ($\mu_{a}$), and (3) the anisotropic factor (g). Specific explanations for scattering factors can be found in the literature.^27^ Light scattering in the volume was performed by combining the Henyey-Greenstein model^28^ with Monte-Carlo simulation. Two review papers^27^^,^^29^ reported the measured data of three scattering factors for most human tissues: when the wavelength is 580 nm (1) g is around 0.9; (2) $\mu_{a}$ is $<0.35\;{\mathrm{mm}}^{-1}$; and (3) $\mu_{s}$ is 20 to $50\;{\mathrm{mm}}^{-1}$. Therefore, in the simulation, g was set at 0.9, $\mu_{a}$ was set at $0.35\;{\mathrm{mm}}^{-1}$, and $\mu_{s}$ was set at $20\;{\mathrm{mm}}^{-1}$ or $50\;{\mathrm{mm}}^{-1}$ for comparison of results. The acceptance NA of the multimode fiber was set at 0.5, which is larger than the biggest NA formed by the collection lens.

Figure 2(b) shows the increase of the collected power by the multimode fiber as its core becomes larger. The decrease of the power as the point source moves to the off-axis field is reasonable. Meanwhile, because the effective aperture of the endomicroscopic tube becomes the limiting factor when signals are scattered to a distance far beyond the FOV, all curves in Fig. 2(b) reach the maximum collection as the fiber core is larger than 1.25 mm. A multimode fiber with 1.5 mm core diameter and 0.5 acceptance NA is therefore selected to collect the maximum amount of signals. The collected power at the largest fiber core is enhanced by a higher scattering coefficient. Figure 2(c) is the change of the collected power as the excitation beam focuses from the volume surface to 160  μm inside. The multimode fiber diameter was set at 1.5 mm for collecting maximum signals. In the case of $\mu_{s}=50\;{\mathrm{mm}}^{-1}$, the power gets reduced as the image depth gets longer. However, for a lower scattering coefficient, i.e., $\mu_{s}=20\;{\mathrm{mm}}^{-1}$, the change is not evident along the depth. As a conclusion, the detection efficiency is increasing when more scattering events take place in the tissue. Besides, for highly scattering tissue, a shorter imaging depth could significantly enhance the collection efficiency. However, simulation results here are limited by two facts: (1) the scattering of the excitation light when being focused into the tissue was not considered and (2) isotropic radiation pattern of the point source is not true for CARS and SHG processes, instead they have a stronger directed characteristic.^30^^,^^31^

## 3 Qualification Results

### 3.1 Optical Performance of the Endomicroscopic Tube

**Fig. 3:**
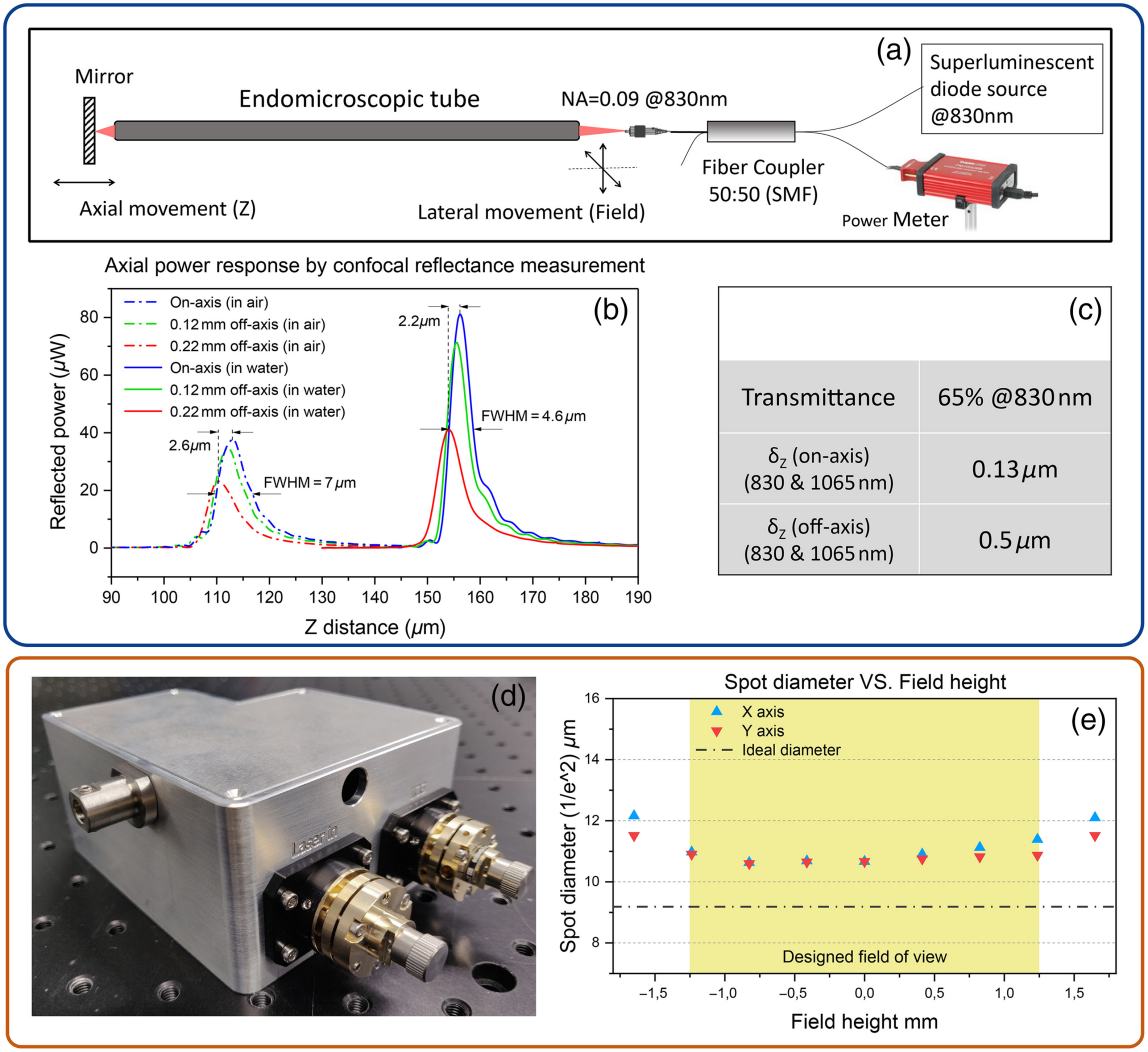
(a) Experimental setup of the confocal reflectance measurement. (b) Axial dependence of the back-coupled power at different field heights and immersion mediums. Solid and dash-dotted lines indicate the working space of the distal end in water and air. Blue, green, and red represent measurement results at different field heights. (c) Other specifications of the endomicroscopic tube measured by the confocal reflectance setup (working space was in air). (d) Picture of an assembled scan-head (fibers were not mounted). (e) Spot size measured in the coupling plane to the endomicroscopic tube as the field height varies. Light in 830 nm was coupled into the hollow-core fiber in the measurement [not shown in panel (d)].

The rigid endomicroscopic tube of 0 deg-view was tested by a confocal reflectance setup as outlined in Fig. 3(a). An SM fiber connected to an 830 nm super luminescent diode (SLD) source was used as a light source to couple the light in the endomicroscopic tube. The back reflected light from a mirror at the distal end was sent back to the same fiber and measured by a detector via a single-mode 2×2 fiber coupler (TW850R5A2, Thorlabs). By moving the mirror in Z direction and the SM fiber in the lateral direction, the axial dependences of the back-coupled power at different field heights were measured. Meanwhile, different immersion mediums (water and air) in the working space were tested, and results are individually shown by the solid lines and dash-dotted lines in Fig. 3(b). For each response function, the peak position indicates the working distance of the current field in that medium. Consequently, the system had a longer working distance in water, and the peak powers for all fields in water were almost doubled compared to the same field in air. The on-axis power response in water shows a smaller full width at half maximum value (axial FWHM: 4.6  μm) than in air and its side lobes near the major peak were less dominant. This is because the optical design was optimized for the use in water medium and, the endomicroscopic tube exhibited less spherical aberration while the working space was in water. Due to a smaller difference of refractive indices, there was also less reflection loss between the water and glass window. The peak shift among different fields related to the field curvature generated by the endomicroscopic tube. From the on-axis to the field edge (220  μm), the focal shift was 2.2  μm in water and 2.6  μm in air, which are smaller than the axial FWHM, so the field curvature can be neglected.

Transmission test and chromatic aberration measurement were also performed with the same setup when the working space was in the air [Fig. 3(c)]. The endomicroscopic tube had a transmittance of 65% at 830 nm. The chromatic focal shifts between 830 and 1065 nm were 0.13  μm for the on-axis field and 0.5  μm for the field edge. Since these values are much smaller than the axial FWHM measured in Fig. 3(b) (7  μm in air), two wavelengths are supposed to coincide well in the longitudinal direction.

### 3.2 Optical Performance of the Scan-Head

Optical performance of the assembled scan-head was assessed by measuring the spot size in scan-head’s output. A beam profiler (SP928, Spiricon) combined with an objective lens (4× DIN, Edmund Optics) was utilized for searching the focal plane and then measuring the beam size. The blue and red triangles in Fig. 3(e) represent the spot size of different fields in the x and y axis, which were achieved by setting two scanning mirrors to different angles. The ideal spot size on the focal was calculated by the paraxial Gaussian beam propagation in Zemax. It was based upon the paraxial ray data of the optical system where no aberration was taken into account, and assumed that the Gaussian beam propagates well within lens apertures.^32^ In the simulation, the incoming beam from the delivery fiber was set the same parameters as the measured data: wavelength 830 nm, beam waist 15  μm, and $M^{2}$ 1.3. Yellow region marks a FOV of 2.4 mm on the coupling plane to the endomicroscopic tube, which conjugates a 430  μm FOV at tube’s distal end. Although the system generates extra aberrations resulting in a larger spot diameter, it behaves stable as the field height increases within the yellow region. Since the Galvo scanner has two scanning mirrors separated by a certain distance, spot sizes on the focal plane in one axis are larger than the other, especially when the field height is beyond the yellow region. The asymmetric tendency in the x axis was caused by components’ misalignment in the assembly.

## 4 Preliminary Endomicroscopic Imaging and fs Laser Ablation Tests

### 4.1 USAF Target Imaging by Confocal Detection Channel

**Fig. 4:**
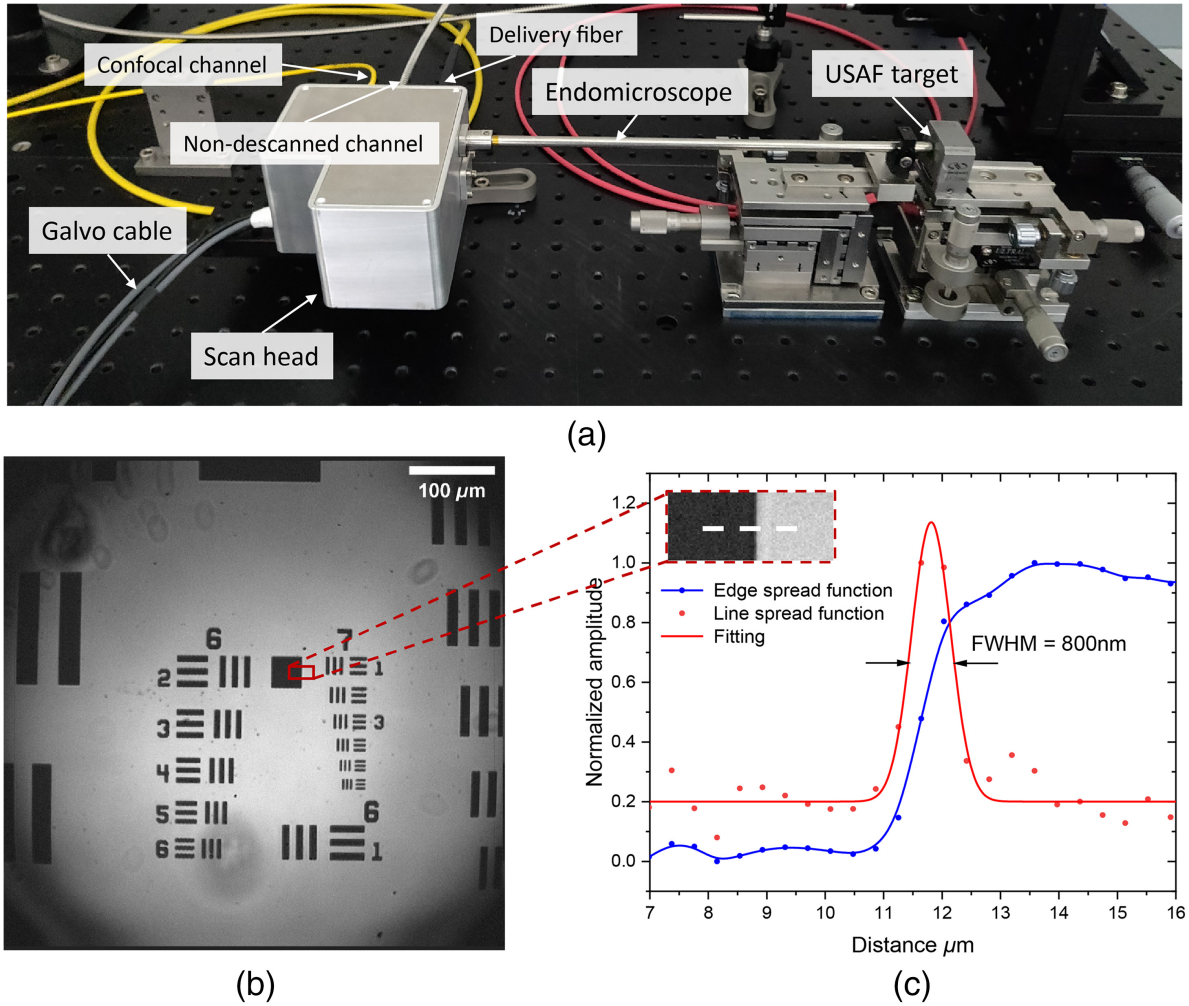
(a) Endomicroscopic device while imaging a USAF target. Fibers for laser delivery, confocal collection, and non-descanned collection are arranged on the same side. (b) Image of the resolution target. Imaging parameters: 1500  px×1500  px; 600 lines per second; 0.36  μm pixel width. The smallest group can be easily resolved. (c) Line spread function of an area near the center of the field. Edge spread function (blue dotted line) is the gray profile along the white dash line. Red points are derivative values of the blue sampling points.

As a preliminary imaging test of the whole system, a resolution target (1951 USAF Resolution Test Targets, Thorlabs) was used as the reflective component for laser confocal imaging. As shown in Fig. 4, the target was mounted on the side of tube’s end, and adjusted to the best focusing position while the working space was in air, i.e., around 115  μm from the glass window. Light from an 830 nm SLD source was coupled in the system through the hollow-core delivery fiber. The reflection signal was transmitted back and collected by the SM fiber (780HP, Nufern) in the ICG confocal channel. A Si-amplified detector (PDA8A/M, Thorlabs) detected signals from the SM fiber for image reconstruction. The Galvo scanner was driven in unidirectional line-scanning mode, for doing that 1500 lines were scanned over 550  μm FOV resulting in 360 nm per pixel size. Bubbles in Fig. 4(b) were caused by some particles on the window surface of the endomicroscopic tube. By analyzing the line spread function of a centered area [Fig. 4(c)], 800 nm resolution in the ICG confocal collection channel was demonstrated.

### 4.2 Resolution Assessment with Fluorescence Beads

**Fig. 5:**
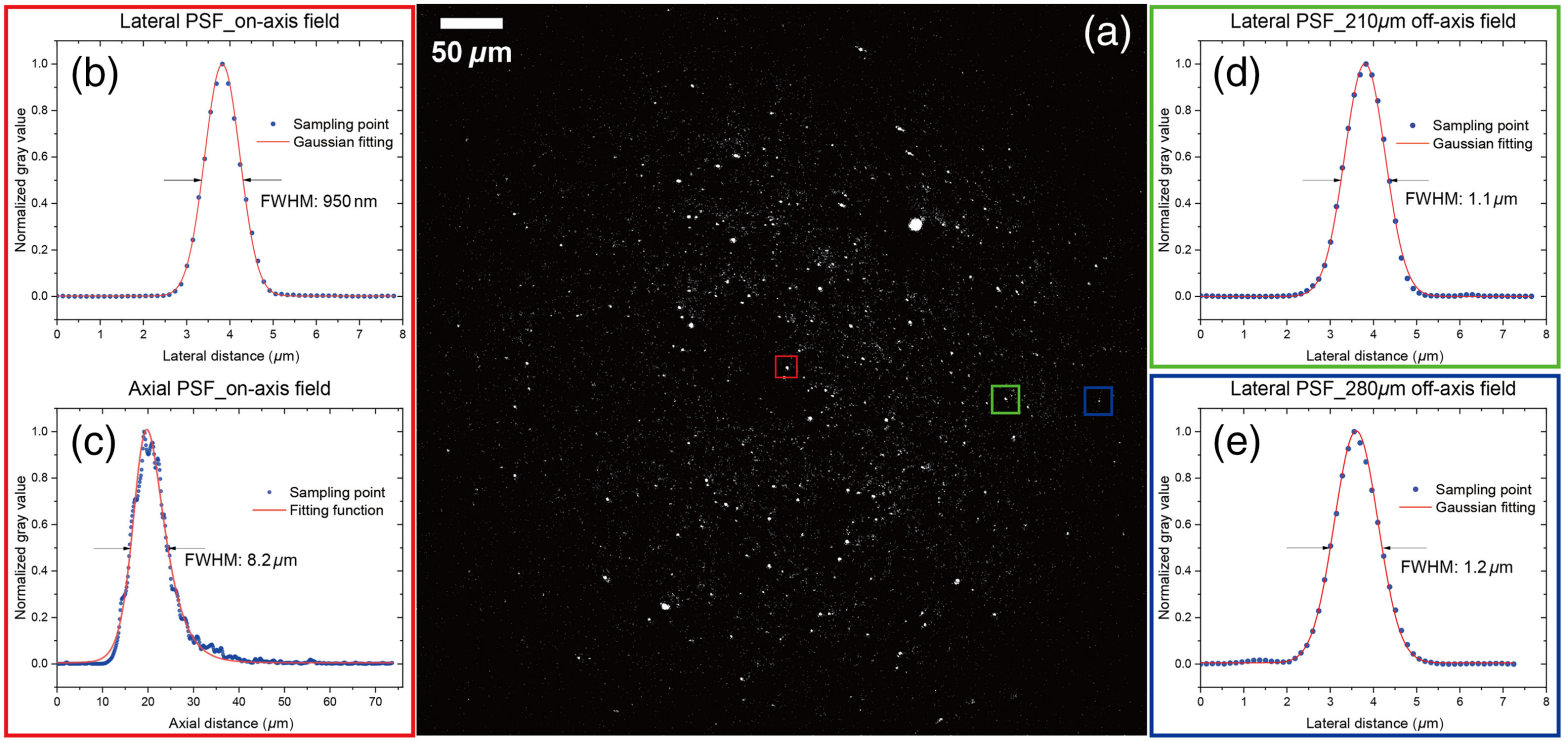
(a) 200 nm fluorescence beads (polystyrene, surface modification by carboxylate) image from TPEF signals, excited by a FOPG pulsed laser for multimodal nonlinear imaging (pump beam at 795 nm and Stokes beam at 1030 nm, 5 MHz repetition rate, 22 mW, and 110 mW average power at the sample, respectively for pump and Stokes). Filter: BP514/3. (b), (d), and (e) Lateral PSF of points framed by red, blue, green squares in panel (a), with pixel size of 0.13  μm in width, 4  μs in dwell time, and frame averaged by eight times. (c) Axial PSF of a point at the central field, i.e., point framed by the red square in panel (a). Stack step is 0.5  μm in z direction. Images were acquired by the ScanImage (Vidrio Technologies).^33^

As a preliminary nonlinear imaging test, 200 nm fluorescence beads (F8811, Thermo Fisher Scientific Inc) were first imaged by probing TPEF signals in a narrow band [Fig. 5(a)]. The immersion medium at the distal end of the endomicroscopic tube was water. To investigate the resolution of different fields, three areas in Fig. 5(a) were chosen for detailed imaging by applying a zoom factor. The FOV of each zoomed image was 70  μm in width. By plotting their lateral PSFs in Figs. 5(b), 5(d), and 5(e) and calculating the relative FWHM, it can be concluded that the system achieves a lateral resolution of ∼1  μm over 560  μm FOV. In the central zoom area, a z-stack was furthermore recorded for characterizing the axial resolution. The movement of the beads sample was achieved by a motorized actuator (Z825B, Thorlabs Inc., United States) with 0.5  μm for each step. The axial profile of the bead in the red square was plotted in Fig. 5(c). It shows an asymmetric distribution because of the spherical aberration produced by the system, which is comparable to the results measured by the axial confocal reflectance setup in Fig. 3(c). Comparing FWHM values of two measurements, the longer FWHM value (8.2  μm) obtained from the axial bead’s profile might be caused by following reasons: (1) the scan-head generated additional aberration; (2) $M^{2}$ of the hollow-core fiber was bigger than 1; (3) different numerical apertures were focused at the distal end; and (4) frame averaging degraded the resolution.

### 4.3 Multimodal Endomicroscopic Imaging of *Ex-Vivo* Human Tissue

Multimodal endomicroscopic imaging of an ICG-contained tissue sample, including single photon fluorescence of ICG and nonlinear imaging (CARS/SHG/TPEF) of endogenous molecular structures, was performed using in combination a CW laser and a pulsed laser. They were employed sequentially in consideration of thermal- and photo-stability of ICG agent.^34^ For ICG fluorescence excitation, a fiber-coupled CW laser diode (781 nm, Point Source Ltd.) at low power was used to reduce bleaching of the dye. For nonlinear imaging, a picosecond Ytterbium-based FOPG (Active Fiber Systems, GmbH), generating a 30 ps pump beam at 795 nm and a 72 ps Stokes beam at 1030 nm with a repetition rate of 5 MHz, was employed. The combination of several modalities allows a morphochemical characterization of the sample: CARS at $2850\;{\mathrm{cm}}^{-1}$ is commonly used to detect methylene groups abundant in lipids and proteins, TPEF allows the detection of auto-fluorophores such as elastin and metabolic components, and SHG highlights the collagen content in the tissue. Instead, ICG localizes to vascular structures after injection because it binds to albumin in the blood. ICG fluorescence emission is peaked in the NIR region, above 800 nm. However, ICG also involves a direct transition from S2 to S0 state as a unique example of the violation of Kasha’s rule, leading to two-photon fluorescence signals in the visible range.^35^ Therefore, a proper detection unit was built to avoid the peak wavelength of S2 - S0 emission (570 to 630 nm)^35^ from falling in the nonlinear modalities’ channels. A detailed description of the detection unit can be found in the Supplementary Material.

The tissue sample was obtained from a patient with squamous cell carcinoma of the head and neck during surgery. The patient received ICG intravenously during surgery according through a standardized protocol.^36^ The sample was preserved by snap freezing in liquid nitrogen, and stored at −80°C until the measurement. The study was approved by the ethics committee of the Jena University Hospital (No. 4291-12/14).

**Fig. 6:**
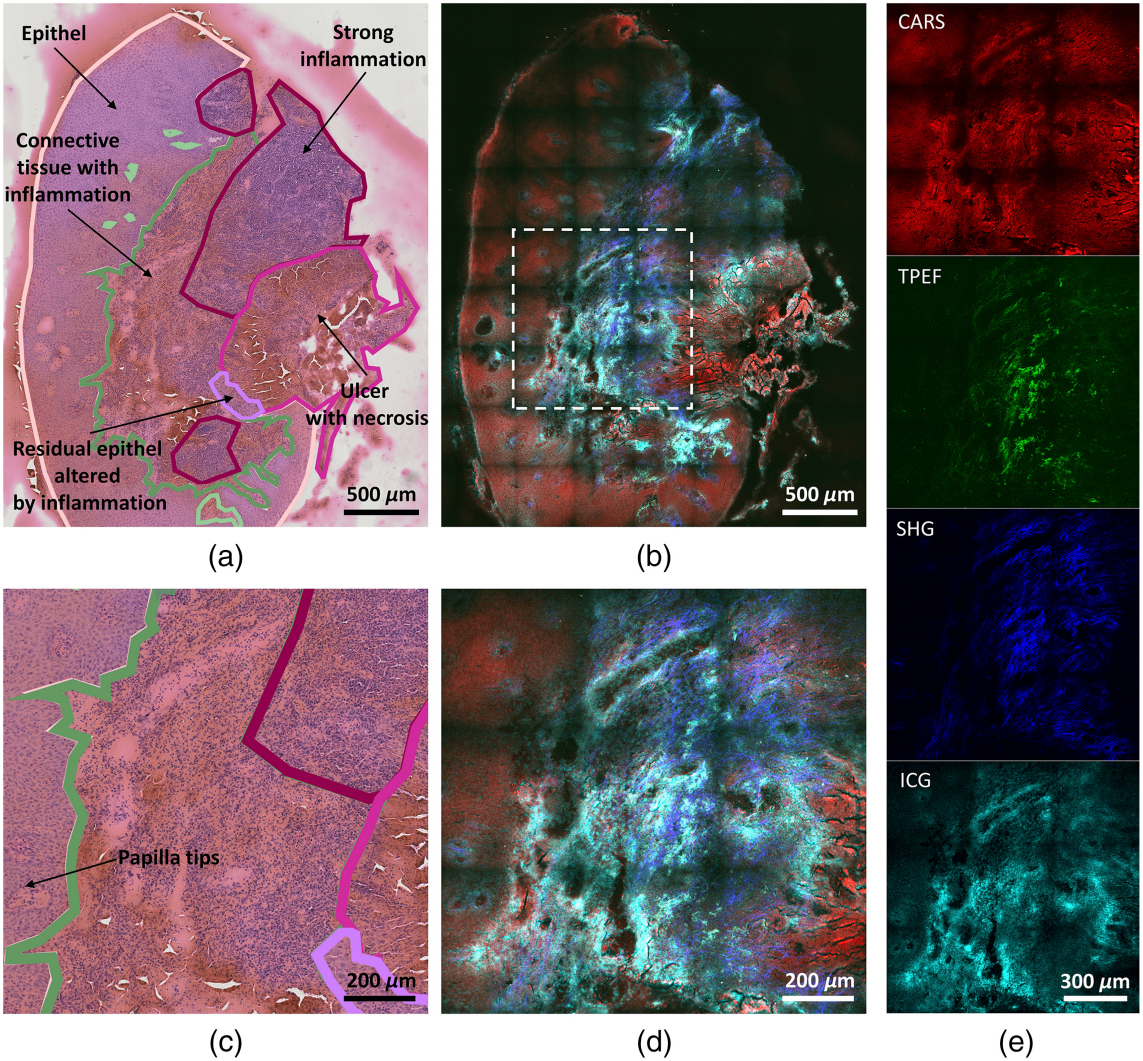
Comparison of H&E stained and multimodal endomicroscopic images of head and neck tissue sample from a patient with squamous cell carcinoma (after ICG injection). (a) H&E stained image with pathohistological annotations. (b) Multimodal overview image of the same sample, created by stitching the 10×10 tiles with 8.5% overlap and cropped to visualize only the sample region. (c) Zoomed H&E stained image of the inset marked in panel (b). (d) Zoomed multimodal image of the inset marked in panel (b). (e) Single modality images of (d). Power at the sample: 0.85 mW (CW laser at 781 nm, for ICG detection), 18.5 mW (FOPG pump beam at 795 nm), and 106 mW (FOPG Stokes beam at 1030 nm). 5 frames averaging for the multimodal images. Color map: red (CARS), green (TPEF), blue (SHG), and cyan (ICG).

A section of 30  μm thickness was cut with a cryotome (Leica Biosystems) and, after the measurements by the endomicroscope, it was stained with hematoxylin and eosin (HE) and submitted to an experienced pathologist for analysis and annotation, as shown in Fig. 6(a).

The measurement routine consisted in an overview acquisition of the sample with 10×10 tiles. Each tile shows a 430  μm FOV recorded with 1200×1200  pixels, 3  μs pixel dwell time, and 5 frames averaging. The procedure was performed once with the CW laser for ICG detection only and subsequently with the pulsed laser for nonlinear imaging. The power of the CW laser at the sample was 0.85 mW, while the pump and Stokes beams power of the FOPG were 18.5 and 106 mW, respectively.

Figure 6(b) shows the overview of the sample as a composite overlay of the four modalities. A selected region of interest (ROI) is shown in Figs. 6(c) and 6(d), as H&E stained image and multimodal composite, respectively. The single four channels of the selected ROI are then shown in Fig. 6(e). The combination of the different imaging modalities reflects the morphological tissue structure. According to the pathohistological annotations in Fig. 6(a) the tissue is divided into an epithelial and stroma part. The ICG image highlights an inflammation in the connective tissue. The higher ICG signals are caused by angiogenesis, one hallmark of inflammation, in which the building of blood vessels is increased. The ICG signal also distinguishes papilla tips in the epithelia [Fig. 6(c)]. These papilla structures are surrounded by the lamina propria, a lymphatic vessel rich layer. TPEF and SHG images show the elastin and collagen content, which are the main structural proteins of the extracellular matrix in the connective tissue. They are not expressed by epithelial tissue. CARS signals show the morphology of tissue section. In this modality, the dense structure of the epithelial tissue can be visualized. Additionally, by contrasting the general tissue area it gives the orientation and the spatial integrity of the structures highlighted by the other modalities. Tissue section without ICG was also measured by three nonlinear modalities, and the results are shown in Fig. S1 in the Supplementary Material.

### 4.4 Proof of Concept of the Femtosecond Laser Ablation Capability

The possibility of performing femtosecond laser ablation was first investigated by studying the transmission and dispersion properties of the whole system when high energy ultrashort pulses are coupled into the delivery fiber. The currently established femtosecond laser ablation devices that are used in ophthalmology usually employ near infrared laser sources between 1040 and 1060 nm,^37^^,^^38^ with sub picosecond pulse durations between 200 and 800 fs, pulse energies in the μJ range and repetition rates from 30 kHz to few MHz.^39^ To have comparable laser characteristics, a femtosecond chirped pulse amplification Ytterbium-fiber laser (Active Fiber Systems GmbH) was used as laser source. It can provide up to 10  μJ laser pulses with a minimum duration around 360 fs at 1032 nm, with a tuneable repetition rate from 190 kHz to 19 MHz. The transmission of the whole system was assessed by measuring the average power before the delivery fiber, after the delivery fiber and after the endomicroscope, i.e., at the sample plane. The overall transmission efficiency of the system is 44% at a fiber coupling efficiency around 80%.

**Fig. 7:**
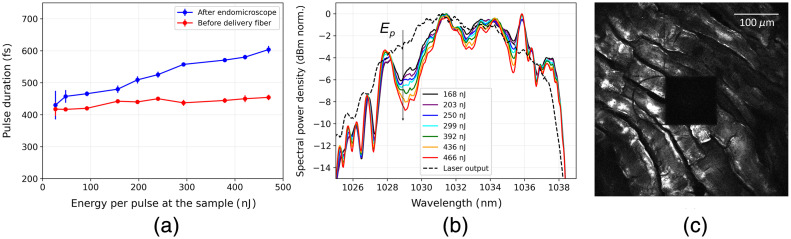
(a) Pulse duration (${\mathrm{sech}}^{2}$ fitting) retrieved from the autocorrelation measurement at different energy per pulse. The red dots refer to the duration of the pulse at the laser output, before the delivery fiber; the blue dots show the values of the pulse duration after the endomicroscope, at the sample plane. The x-axis values are the energy per pulse at the sample plane. (b) Pulse spectrum for different energy per pulse. They are the normalized dBm spectra of the pulse after the endomicroscope, measured for increasing energy. The dashed black line is the reference spectrum at the laser output, measured when the energy at the sample is around 450 nJ. The formation of holes in the spectrum for high energies is clear. (c) Square area ablated on a chicken meat slice with 290 nJ pulses, 5 scans per focal plane at four different focal planes with step size of 4  μm. The figure is a CARS image at $2850\;{\mathrm{cm}}^{-1}$ recorded in ScanImage (Vidrio Technologies).^33^

The laser system also includes a pulse compressor whose dispersion parameters can be varied, allowing pre-chirping of the pulse before the laser output. This functionality was exploited to minimize the pulse duration and optimize the pulse temporal shape at the distal end. The dispersion properties of the system were then assessed by measuring the pulse duration and the spectrum before the delivery fiber and after the endomicroscope. The autocorrelation function of the pulses was measured with an autocorrelator (PulseCheck, A.P.E.) varying the energy per pulse in a range from 20 to 500 nJ at a fixed repetition rate of 190 kHz. The maximum limit around 500 nJ was chosen to avoid possible damages in the endomicroscope’s optics and because an effective ablation at lower energy per pulse was already proven with several tests on biological samples.^23^ Similar laser parameters were also used in previous works.^6^ The pulse temporal shape was retrieved directly from the ${\mathrm{sech}}^{2}$ fit in the A.P.E. software. Figure 7(a) shows the behaviour of the pulse duration before and after the whole system. An increase of the pulse duration was observed for higher pulse energies, demonstrating possible nonlinear dispersion effects in the delivery fiber and in the endomicroscopic system itself. To investigate the origin of such temporal broadening, the pulse spectrum before and after the system was measured using an optical spectrum analyzer (Yokogawa AQ6370C), varying the pulse energy in the same range. Figure 7(b) illustrates how the spectral shape changes when the energy increases. The formation of modulations at the edges of the spectrum indicates self-phase modulation. Nevertheless, the temporal broadening of the pulse through the system is below 200 fs at 470 nJ per pulse. Herein, we conclude that the system can efficiently transmit 400 fs pulses without a considerable broadening. To further confirm the ablation capability of the system, a square area was ablated on a 50  μm chicken meat tissue slice and the CARS channel of our detection system was chosen to assess the extent of the ablation crater, as shown in Fig. 7(c). The ablation region has been created by scanning the laser with a speed of 6.3  mm/s covering an area of 116  μm×116  μm.^22^^,^^40^ After five consecutive scans on the focal plane, the sample was moved towards the probe tip with 4  μm steps, repeating the five scans procedure for each focal plane. By moving the specimen toward the probe tip, the laser can be focused within the tissue, and the ablated volume can be increased. The ablation was performed with 290 nJ pulses at the lowest possible repetition rate of 190 kHz to reduce the possibility of accumulation effects between pulses. The use of ultrashort pulses with a high energy, in combination with the tight focusing of the 0.5 NA micro objective, results in a plasma-induced ablation effect. Unlike CW or long pulses (nanoseconds or more) ablation in which linear absorption of the irradiated energy is the main mechanism leading to a thermal ablation process, the use of femtosecond pulses involves nonlinear absorption processes, and the ablation mechanism is generally referred to in the literature as optical breakdown.^18^^,^^41^ To reach optical breakdown and achieve ablation, the laser fluence on the sample plane must exceed a certain threshold. In Fig. 7(c), we use a fluence up to one order of magnitude higher than that observed as a threshold in similar biological tissues.^42^

## 5 Conclusion and Discussion

In this work, a portable endomicroscopic device integrating single-photon ICG and nonlinear imaging has been designed, built, and characterized. By optimizing the aberration and vignetting in the optical design, the device imaged a maximum of 650  μm FOV in the confocal imaging test and achieved 1  μm resolution by imaging fluorescence micro-beads. An ICG-contained tissue sample was imaged by the device. Four imaging modalities successfully display complementary contrasts for different contents, and the results are comparable to the pathohistological annotations of the sample after H&E staining process. Video 1 demonstrates a 3.3 frame per second (fps) nonlinear imaging of a chicken meat sample in *ex-vivo*. Although its resolution is quite low compared to Fig. S1 in the Supplementary Material, it shows a large FOV (around 700  μm) without apparent delay in the movement, which is not far from real-time imaging. To further increase the imaging speed in full FOV, one method is to replace one of the Galvo mirrors with a resonant scanning mirror, so that the frame rate can be increased to around 25 fps.^43^ Furthermore, to preserve the image quality, considering the current laser parameters, laser source with significantly higher repetition rates and shorter pulse lengths are needed in the future.

System’s capability of transmitting high-energy fs pulses for laser ablation was proved by ablating a 116  μm square on a chicken meat sample. Nonlinear pulse broadening was observed while measuring the pulse duration before and after the system, and around 600 fs duration of a 470 nJ energy pulse was measured at the distal end. However, the ablation test in this work is still in its early stages, and further investigation of the ablation process and efficiency in biological tissues is needed.

Overall, this new device exhibits great potential for real-time tissue diagnosis during surgery. Fast scanning mode (3.3 fps) with a low resolution allows the user to scan the full FOV (>650  μm) for identifying an ROI, which can then be imaged in high resolution by two options, either having full FOV but with a low scanning rate, or having a fast scanning rate but with a smaller FOV (around 100  μm×100  μm). *In-vivo* imaging tests have not performed yet, but the idea is to mount the device onto a surgical robotic arm and navigate by a conventional bright-field endoscope. By making the tip of the endomicroscope touch the surface of suspicious tissue area, motion artifacts can be much reduced. Further motion correction is even possible with computational methods.^44^

In the future, more investigations are needed to understand the applications of this device under clinical conditions.
