# Supplementary material for: Design and test of a rigid endomicroscopic system for multimodal imaging and femtosecond laser ablation
Source: J Biomed Opt. 2023 Jun 28;28(6):066004. doi: 10.1117/1.JBO.28.6.066004 (PMC10306116; doi:10.1117/1.JBO.28.6.066004)
Supplement: Supplementary file 1 [file JBO_028_066004_SD001.pdf]

Supplementary information for

Design and test of a rigid endomicroscopic system for multimodal imaging and femtosecond laser ablation

Chenting Lai,[a] Matteo Calvarese,[b] Karl Reichwald,[a] Hyeonsoo Bae,[b,c] Mohammadsadegh Vafaeinezhad,[b,c] Tobias Meyer-Zedler,[b,c] Franziska Hoffmann,[d] Anna Mühlig,[d] Tino Eidam[e], Fabian Stutzki[e], Bernhard Messerschmidt,[a] Herbert Gross,[f] Michael Schmitt, [c] Orlando Guntinas-Lichius,[d] and Jürgen Popp,[b,c]

[a]GRINTECH GmbH, Jena, Germany
[b]Leibniz Institute of Photonic Technology, Member of Leibniz Health Technologies, Member of the Leibniz Centre for Photonics in Infection Research (LPI), Jena Germany
[c]Institute of Physical Chemistry (IPC) and Abbe Center of Photonics (ACP), Friedrich Schiller University Jena, Member of the Leibniz Centre for Photonics in Infection Research (LPI), Jena, Germany
[d]Department of Otorhinolaryngology, Jena University Hospital, Jena Germany
[e]Active Fiber Systems GmbH, Jena Germany
[f]Fraunhofer Institute for Applied Optics and Precision Engineering, Jena Germany

Detection unit in the multimodal endomicroscopic imaging

For separating signals from the tissue into different wavelengths, a proper detection unit including many filters and four photomultiplier tubes (PMTs, Hamamatsu) was built. The output of the SM fiber for ICG detection was collimated by using a fiber collimator and then filtered to suppress the laser reflection. Following filters were employed: LP800, LP808, LP830, BP850/40, and SP950. The nonlinear signals coming from the multimode fiber were collimated by a lens 25 mm in focal length and filtered by a SP700. Then, two dichroic mirrors were used to spatially separate them into three wavelength ranges of interest: (1) dichroic mirror LP605 transmits only the CARS signal around 650nm to a BP650/40 and subsequently to an f = 30 mm lens which focuses the beam on the PMT. The reflected signals are further split by a dichroic LP495, and the TPEF spectral range is reflected and directed to an f = 40 mm lens and a PMT, while the SHG is transmitted, filtered by a BP514/3 and focused by an f = 30 mm lens onto the third PMT.

Multimodal endomicroscopic image of the tissue sample

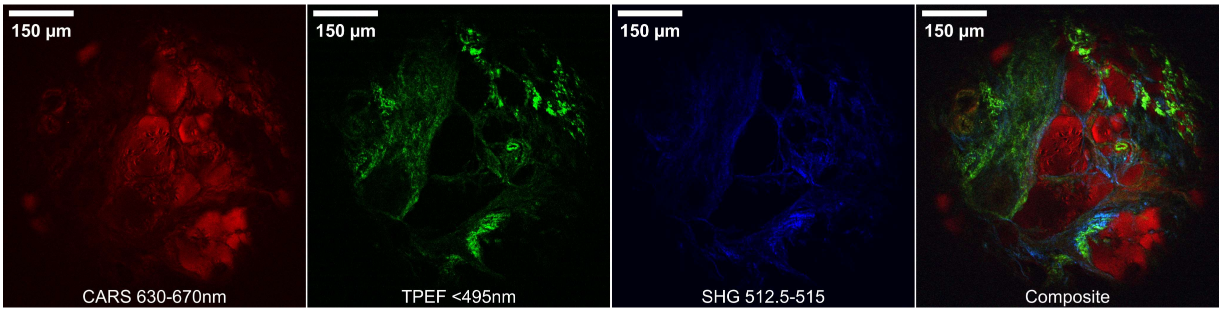

Fig. S1 Endomicroscopic multimodal images of a head and neck tissue from a patient not receiving ICG. CARS (red), TPEF (green), and SHG (blue) display different contrasts for methylene groups abundant in lipids and protein, auto-fluorophore and collagen content, respectively. Three modalities were excited by a ps pulsed laser (Pump: 795 nm / 30 ps / 22 mW; Stokes: 1030 nm / 72 ps / 100 mW) and acquired simultaneously by 2048×2048 pixels with 6 µs in dwell time, and frame averaged by 5 times. Images were acquired by ScanImage (Vidrio Technologies) [33]. The study was approved by the ethics committee of the university hospital Jena (No. 4291-12/14).

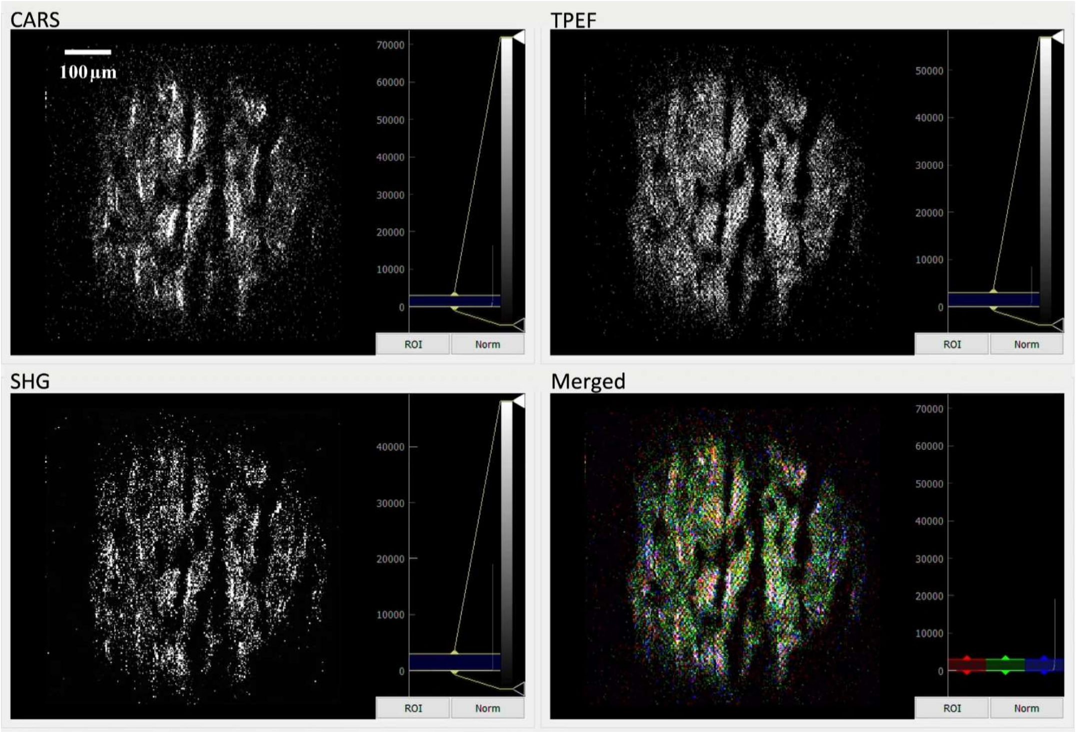

Fig. S2 Endomicroscopic live imaging of a chicken meat sample in ex-vivo, CARS (red), TPEF (green), and SHG (blue). To achieve a fast scanning of about 3.3 fps over a FOV of around 700 µm, the number of pixels was decreased to 250x250 pixels, with a pixel dwell time of 3 µs. The imaging was performed with a custom-made and python-based software, that utilizes the Scope Foundry (http://www.scopefoundry.org/) framework. (MP4, 8.5 MB).
